# Routine saliva testing for the identification of silent coronavirus disease 2019 (COVID-19) in healthcare workers

**DOI:** 10.1017/ice.2020.1413

**Published:** 2021-01-11

**Authors:** Kevin Zhang, Affan Shoukat, William Crystal, Joanne M. Langley, Alison P. Galvani, Seyed M. Moghadas

**Affiliations:** 1 Faculty of Medicine, University of Toronto, Toronto, Ontario, Canada; 2 Center for Infectious Disease Modeling and Analysis (CIDMA), Yale School of Public Health, New Haven, Connecticut, United States; 3 Canadian Center for Vaccinology, Dalhousie University, IWK Health Centre and Nova Scotia Health Authority, Halifax, Nova Scotia, Canada; 4 Agent-Based Modelling Laboratory, York University, Toronto, Ontario, Canada

**Keywords:** COVID-19, testing, nasopharyngeal, saliva, case detection, outbreak

## Abstract

**Objective:**

Current COVID-19 guidelines recommend symptom-based screening and regular nasopharyngeal (NP) testing for healthcare personnel in high-risk settings. We sought to estimate case detection percentages with various routine NP and saliva testing frequencies.

**Design:**

Simulation modeling study.

**Methods:**

We constructed a sensitivity function based on the average infectiousness profile of symptomatic coronavirus disease 2019 (COVID-19) cases to determine the probability of being identified at the time of testing. This function was fitted to reported data on the percent positivity of symptomatic COVID-19 patients using NP testing. We then simulated a routine testing program with different NP and saliva testing frequencies to determine case detection percentages during the infectious period, as well as the presymptomatic stage.

**Results:**

Routine biweekly NP testing, once every 2 weeks, identified an average of 90.7% (SD, 0.18) of cases during the infectious period and 19.7% (SD, 0.98) during the presymptomatic stage. With a weekly NP testing frequency, the corresponding case detection percentages were 95.9% (SD, 0.18) and 32.9% (SD, 1.23), respectively. A 5-day saliva testing schedule had a similar case detection percentage as weekly NP testing during the infectious period, but identified ~10% more cases (mean, 42.5%; SD, 1.10) during the presymptomatic stage.

**Conclusion:**

Our findings highlight the utility of routine noninvasive saliva testing for frontline healthcare workers to protect vulnerable patient populations. A 5-day saliva testing schedule should be considered to help identify silent infections and prevent outbreaks in nursing homes and healthcare facilities.

The novel coronavirus disease 2019 (COVID-19) has led to a devastating global pandemic.^1^ The burden of disease has been disproportionately high in some healthcare settings and long-term care facilities, with case fatality rates exceeding 30%.^2,3^ Most COVID-19 cases among healthcare workers (HCWs) are the result of community exposure,^4^ posing a potential risk of transmission to immunocompromised individuals and those at higher risk of developing adverse clinical outcomes.^5–8^ Modeling analyses show that rapid case identification of infected persons is critical to interrupt transmission, especially for infectious cases without clinical symptoms.^9^

Current case detection approaches in healthcare settings rely on symptom-based screening and nasopharyngeal (NP) testing for symptomatic or exposed HCWs.^10,11^ Some jurisdictions have recommended routine biweekly or weekly NP testing for frontline HCWs in facilities at risk of severe COVID-19 outbreaks, such as nursing homes.^10,11^ The NP test to detect nucleic acid or antigen, however, is relatively invasive and requires trained personnel for sample collection. On the other hand, saliva tests can be self-administered and, therefore, are easier to implement, potentially more acceptable, and reduce the need for personal protective equipment (PPE) during sample collection.^12^ Since up to 80% of COVID-19 cases are mild or asymptomatic^13^ and, therefore, might be missed by symptom-based screening, testing of asymptomatic HCWs could increase detection and prevent transmission during the highly infectious presymptomatic period.^9,14^ An easy-to-administer saliva test could be a more feasible tool to conduct higher frequency testing to curtail silent transmission. A recent modeling study suggests that at least 33% of silent infections must be identified and isolated in the presymptomatic or asymptomatic stage of the disease to enable outbreak control, even when all symptomatic cases are immediately isolated.^9^

Given the importance of testing in preventing onward transmission in healthcare settings, we sought to estimate case detection percentages using reverse-transcriptase polymerase chain reaction (RT-PCR) testing of NP and saliva samples and to ascertain the frequency of testing that may be required to control outbreaks.

## Methods

We simulated a routine testing program with various frequencies of NP and saliva tests over 150 days. In our analysis, we only included individuals who went on to develop a symptomatic course of disease. To estimate case detection percentages, we first constructed a sensitivity function 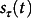 to map the infectiousness profile of symptomatic COVID-19 cases^14,15^ to the reported percent positivity of NP RT-PCR tests after symptom onset.^16^ The infectiousness profile (Appendix Fig. A1 online) was extracted from computer code provided in previous studies that utilized maximum likelihood and optimization methods.^14,15^ The mapping was performed by fitting the sensitivity function to the publicly available percent positivity data of 209 COVID-19 patients for 26 days after the start of symptoms, including the day of symptom onset.^16^ The sensitivity function, expressed as the product of Hill and Gompertz functions, is given by the following equations:
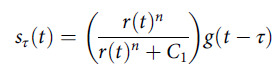


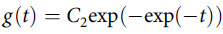



where 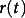 is the average infectiousness profile over time 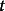, 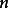 is the Hill coefficient, 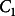 is the Hill saturation constant, and 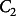 is the Gompertz asymptote level. The parameter 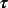 indicates the start of infectiousness, which was assumed to be 1 day after infection within the incubation period. For each infected individual, the incubation period was sampled independently from a log-normal distribution, with parameters 1.434 (shape) and 0.661 (rate), having a mean of 5.2 days.^17^

We fitted the sensitivity function using a least-squares method, and we obtained time-dependent NP RT-PCR sensitivities for different values of 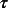, which determined the probability of being detected at the time of an NP test. Given the timelines of infectiousness profile (Appendix Fig. A1 online), we considered a detection period from the start of infectiousness to 15 days after symptom onset as clinically relevant for disease transmission. The case-detection percentage was then calculated as the average probability of all individuals being identified in at least 1 test within their infectious period. To determine the case-detection percentage with a saliva test, we used recent empirical studies for the estimates of saliva testing sensitivity in the range of 70%–97%.^18–20^ Since viral loads in saliva samples have been shown to be comparable to those of NP samples over time,^21–23^ we applied this range to the normalized sensitivity curves of NP testing and determined the temporal sensitivity of a saliva test (Appendix Fig. A2 online). Normalization was done by dividing each point on the fitted NP sensitivity curve by its maximum estimated sensitivity over time. Further details of the model implementation are provided in the Appendix (online).

To derive the distributions for mean case-detection percentages during the infectious period and the presymptomatic stage, we ran 500 independent Monte Carlo simulations by introducing 100 infections on each day for each simulation. The generated distributions were then compared using the Mann-Whitney U test. We conducted this analysis to ascertain the frequencies of testing needed to identify at least one-third, one-half, and two-thirds of silent infections during the presymptomatic stage.

### Ethics approval

This research was based on publicly available data^14–16^ and therefore did not require ethics approval.

### Data sharing

The computational model with parameter values and data pertaining to the simulation study are freely available (https://github.com/affans/npt-saliva-testing).

## Results

### Impact of routine testing on infectious case identification

**Fig. 1.**
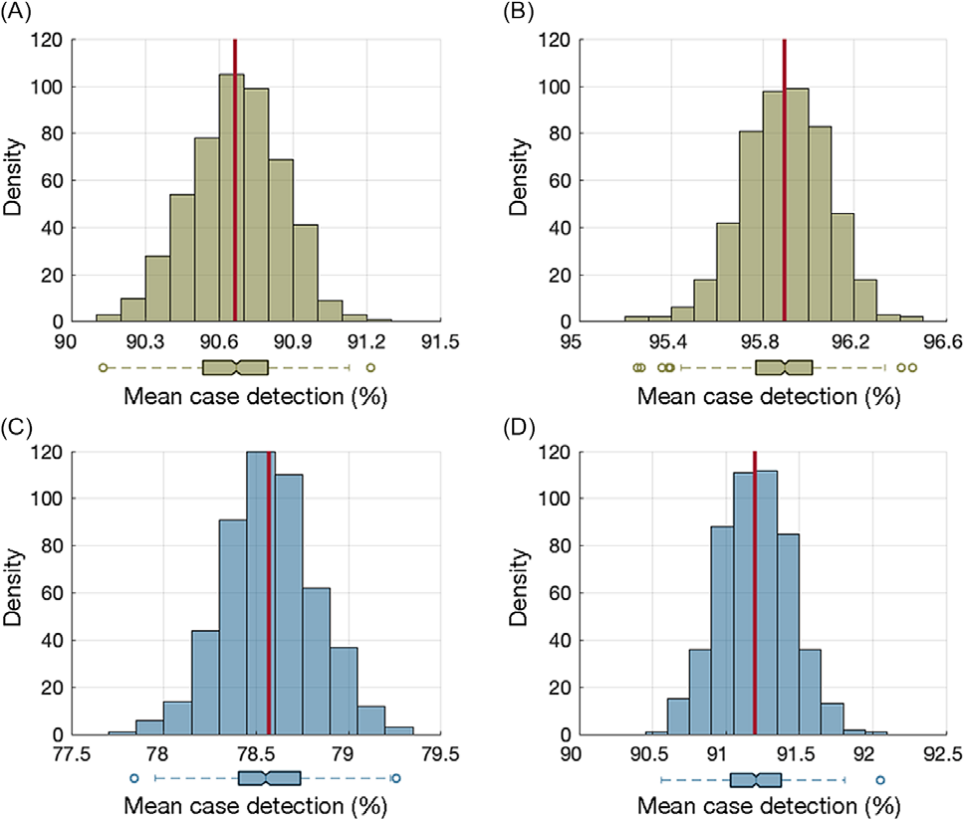
Distribution of mean case-detection percentages during the infectious period using biweekly nasopharyngeal (A) and saliva (C) testing. Distribution of mean case-detection percentages during the infectious period using weekly nasopharyngeal (B) and saliva (D) testing. The red line indicates the mean of the distribution, and the box plot represents the interquartile range (IQR) with whiskers extending the range from minimum (25th percentile minus 1.5 IQR) to maximum (75th percentile plus 1.5 IQR). The density on the *y*-axis is the number of experiments from 500 iterations (Monte-Carlo simulations) that resulted in a mean case detection shown on the *x*-axis.

Biweekly NP testing, once every 2 weeks, identified, on average, 90.7% (SD, 0.18) of cases during the infectious period (Fig. 1A). With a weekly NP testing schedule, the case-detection percentage was 95.9% (SD, 0.18) (Fig. 1B). In total, 81.2% of individuals were detected by the first NP test, irrespective of the testing frequency. Biweekly saliva testing identified a mean of 78.6% (SD, 0.24) of cases during the infectious period (Fig. 1C). When the frequency of saliva testing increased to a weekly schedule, the case-detection percentage was 91.2% (SD, 0.24) (Fig. 1D). With saliva testing, the detection percentage for the first test was 67.8%, irrespective of the testing frequency.

An 8-day saliva testing frequency was required to identify a similar percentage of infectious cases as with NP testing every 2 weeks, with no significant difference between the distributions of mean detection percentages in the 2 tests (Mann-Whitney U test, *P* = .33). A frequency of 5-day saliva testing had a similar infectious case-detection percentage compared to weekly NP testing, with no significant difference in the distributions of mean detection percentages between the 2 tests (Mann-Whitney U test, *P* = .16).

### Impact of routine testing on presymptomatic case identification

**Fig. 2.**
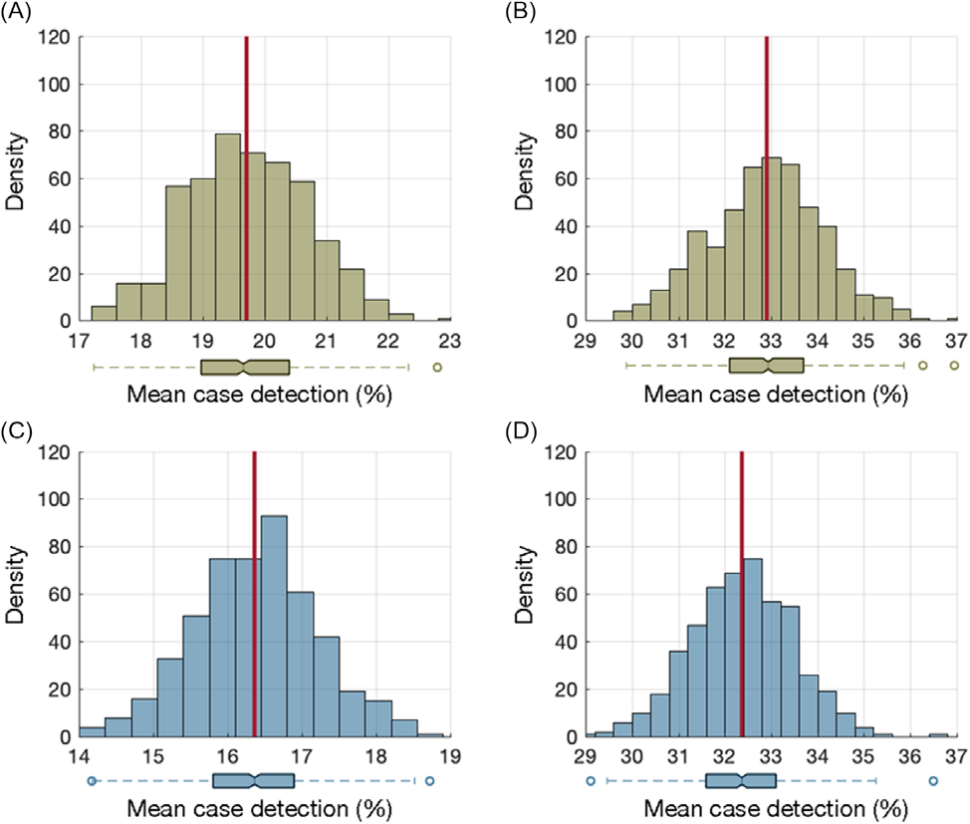
Distribution of mean case-detection percentages during the presymptomatic stage using biweekly nasopharyngeal (A) and saliva (C) testing. Distribution of mean case-detection percentages during the presymptomatic stage using weekly nasopharyngeal (B) and saliva (D) testing. The red line indicates the mean of the distribution, and the box plot represents the interquartile range (IQR) with whiskers extending the range from minimum (25th percentile minus 1.5 IQR) to maximum (75th percentile plus 1.5 IQR). The density on the *y*-axis is the number of experiments from 500 iterations (Monte-Carlo simulations) that resulted in a mean case detection shown on the *x*-axis.

Biweekly NP testing identified an average of 19.7% (SD, 0.98) of cases during the presymptomatic infectious stage (Fig. 2A). With a weekly NP testing schedule, the mean presymptomatic case-detection percentage was 32.9% (SD, 1.23) (Fig. 2B). For saliva testing, the mean case-detection percentages during the presymptomatic stage were 16.4% (SD, 0.83) and 32.4% (SD, 1.10) for biweekly and weekly schedules, respectively (Fig. 2C, 2D). A 5-day saliva testing schedule, while detecting a similar percentage of cases as weekly NP testing during the infectious period, identified a mean of 42.5% (SD, 1.10) of presymptomatic cases, which was significantly different (Mann-Whitney U test, *P* < .001) and ~10% higher than weekly NP testing.

A routine NP testing frequency of at least once every 6 days, 4 days, and 2 days was required for the case-detection percentage during the presymptomatic stage to exceed 33%, 50%, and 67%, respectively. The same saliva testing frequencies would be required to exceed presymptomatic case-detection percentages of 33%, 50%, and 67%, respectively.

## Discussion

Our results show that routine NP testing every 2 weeks or every week, as recommended by some jurisdictions for frontline HCWs,^10,11^ would lead to a significant percentage of undetected silent COVID-19 cases, indicating that institutional outbreaks could occur even in the presence of symptom-based screening.^9^ Recent studies suggest that a significant portion of disease transmission occurs prior to symptom onset,^14,24,25^ highlighting the importance of early detection. Given the practical considerations with NP testing, noninvasive saliva testing presents an attractive alternative for improving case detection with increased testing frequency.^26,27^ Moreover, despite a higher sensitivity, the more invasive NP test did not reduce the required frequency of testing to identify at least 33%, 50%, and 67% of cases in the presymptomatic stage.

Until vaccines are available, healthcare settings and long-term care facilities remain vulnerable to outbreaks that could be seeded through silent transmission by asymptomatic or presymptomatic HCWs. Adherence to public health measures, behavioral interventions, and standard and additional precautions will be essential. Routine testing is an additional intervention that could, along with early case detection of infected HCWs, prevent the introduction of COVID-19 to healthcare settings.^21^ NP testing, while more sensitive compared to saliva testing, is relatively invasive and requires trained personnel to sample individuals, making frequent NP testing impractical for large-scale implementation. For example, a recent study suggests that a testing frequency of every 2 days with a test sensitivity >70% would be needed to prevent outbreaks in postsecondary settings.^28^ Given the high frequency of testing required to detect a sufficient number of silent infections to prevent outbreaks, compliance rates would likely be higher with a noninvasive saliva test.

Our study was based on the assumption of infection a priori; therefore, we did not estimate false positive rates. Given the high specificity of NP and saliva testing estimated at 99.93% (90% CI, 99.77%–99.99%) and 99.96% (90% CI, 99.85%–100.00%), respectively,^18^ false-positive rates would vary depending on the test frequency but are likely to remain <2%. For instance, if tests are done every 2 weeks, with a maximum of 3 tests conducted during the infectious period, 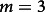, the false-positive rate could reach 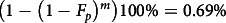 for an upper-bound test false positivity 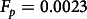 (given a specificity of 99.77%). However, this situation may still lead to a slightly higher rate of self-isolation than necessary compared to a test with perfect specificity because current guidelines recommend that HCWs be excluded from work for 14 days following a known exposure or positive test.^10,11,29^ In our analysis, we only included individuals who went on to develop a symptomatic course of disease. However, given that recent studies have shown similar viral loads for asymptomatic and symptomatic cases,^30,31^ we expect that our case-detection estimates would be applicable for detecting asymptomatic individuals during the infectious period. We also did not model the effect of contact tracing which would readily identify individuals for testing based on known exposures and impose self-isolation if test results are available in a timely manner. In a real-life setting, when contact tracing is combined with routine testing and appropriate referrals are made to a more sensitive NP test as required, the effectiveness of a routine testing strategy would be enhanced. Finally, to evaluate the independent impact of a routine testing strategy, we did not consider other mitigation measures.

Our findings highlight the importance and utility of routine noninvasive saliva testing for frontline HCWs to protect vulnerable patient populations. Coupled with contact tracing and infection prevention and control measures, a 5-day routine saliva testing schedule presents an attractive screening method to reduce the risk of outbreaks in healthcare settings.

## Supplementary material

For supplementary material accompanying this paper visit https://doi.org/10.1017/ice.2020.1413. click here to view supplementary material
